# Spatial and Directional Variation of Growth Rates in *Arabidopsis* Root Apex: A Modelling Study

**DOI:** 10.1371/journal.pone.0084337

**Published:** 2013-12-18

**Authors:** Jerzy Nakielski, Marcin Lipowczan

**Affiliations:** Department of Biophysics and Morphogenesis of Plants, University of Silesia, Katowice, Poland; University of Antwerp, Belgium

## Abstract

Growth and cellular organization of the *Arabidopsis* root apex are investigated in various aspects, but still little is known about spatial and directional variation of growth rates in very apical part of the apex, especially in 3D. The present paper aims to fill this gap with the aid of a computer modelling based on the growth tensor method. The root apex with a typical shape and cellular pattern is considered. Previously, on the basis of two types of empirical data: the published velocity profile along the root axis and dimensions of cell packets formed in the lateral part of the root cap, the displacement velocity field for the root apex was determined. Here this field is adopted to calculate the linear growth rate in different points and directions. The results are interpreted taking principal growth directions into account. The root apex manifests a significant anisotropy of the linear growth rate. The directional preferences depend on a position within the root apex. In the root proper the rate in the periclinal direction predominates everywhere, while in the root cap the predominating direction varies with distance from the quiescent centre. The rhizodermis is distinguished from the neighbouring tissues (cortex, root cap) by relatively high contribution of the growth rate in the anticlinal direction. The degree of growth anisotropy calculated for planes defined by principal growth directions and exemplary cell walls may be as high as 25. The changes in the growth rate variation are modelled.

## Introduction

The symplastic growth, typical for plant tissue, means the coordinated growth of cells during which mutual contacts between neighbouring cells are preserved [1,2]. Such growth is regarded as continuous [3,4], its mathematical description assumes that the displacement velocity, **V**, of material elements of the organ is a continuous and differentiable function of position [5,6].

**Figure 1:**
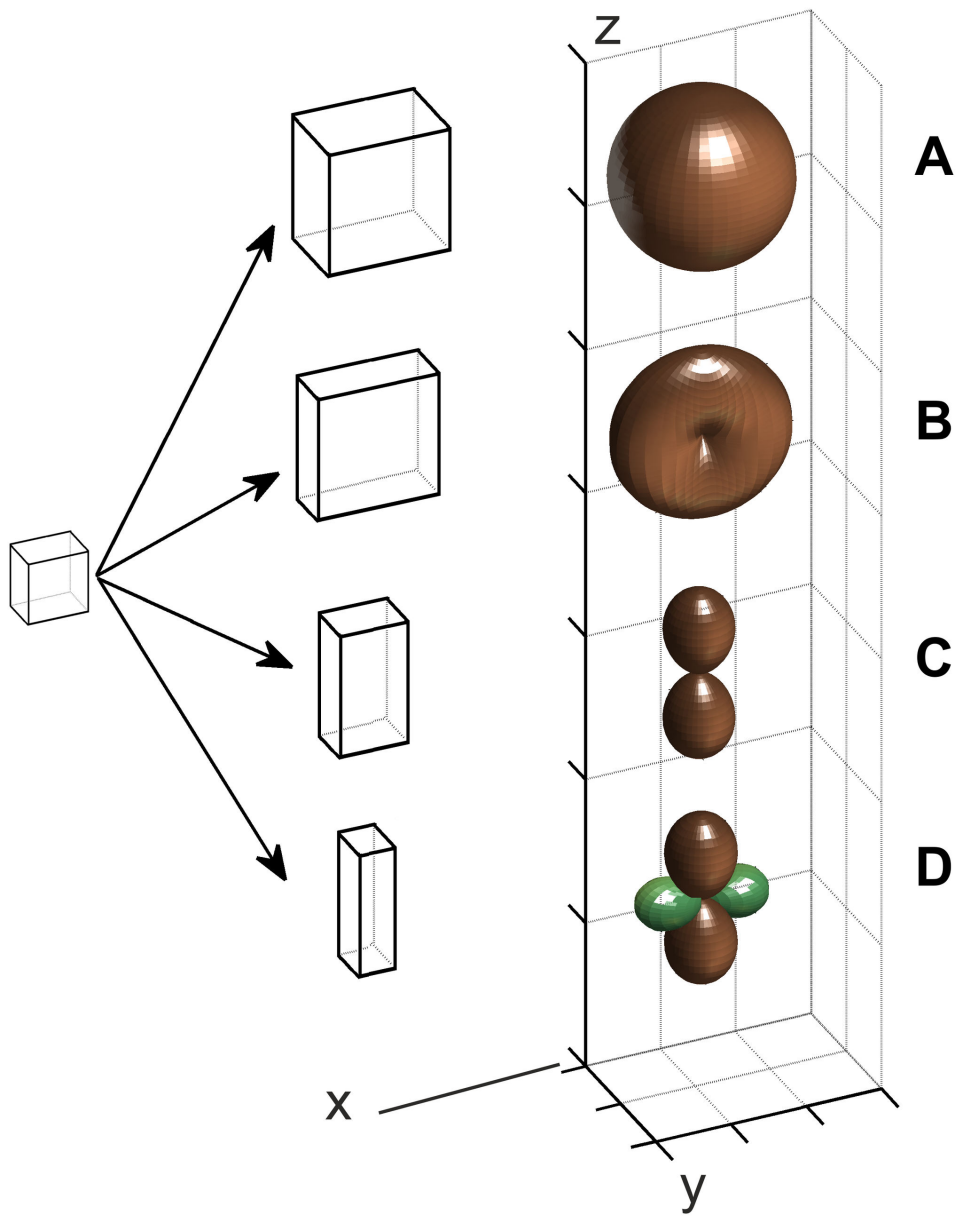
The R_l_ indicatrices representing various growth at a point: isotropic (A) and anisotropic (B-D): (B) symmetry with respect to *y*, i.e. the R_l_ along each direction in *xz* plane is the same, (C) pure elongation along *z*, i.e. there is no growth in *xy* plane, (D) elongation along *z* with contraction (green) along *x*. The scheme on the left shows deformation of the exemplary cell resulting from each growth. In every case R_l_ in a considered direction is proportional to the distance from the calculation point to the indicatrix surface along this direction; the growth rate along *z* axis is always the same.

A measure of growth at a point is the relative elemental rate of the linear growth, R_l_ [6,7], The R_l_ for the direction e_s_ is defined by the equation [5]: R_l(s)_ = (gradV⋅e_s_)⋅e_s_ where e_s_ is the unit vector of the direction and each dot means a scalar product. As this quantity may change with a direction [5,8], values of R_l_ at a point obtained for many e_s_ are arranged into the 3D surface (Figure 1), called indicatrix [9,10]. In such representation R_l_ for particular e_s_ is proportional to a distance from the point to the surface along this direction. For locally isotropic growth, the indicatrix is a sphere (Figure 1A). Indicatrices describing anisotropic growth have various shapes (Fig. B-D). They are plotted using the rule that positive R_l_ is for enlargement, whereas negative (green in Figure 1D) - for contraction [5,8].

The definition of R_l_ includes grad**V** which is the second rank operator [11]. That is why, a field of growth rates of the organ is of a tensor type [3,12]. Such field can be conveniently generated with the aid of the growth tensor (GT), calculated either from grad**V** or as a covariant derivative of **V** [5]. If **V** is determined on the basis of empirical data, the field of growth rates obtained in this way can be assumed as a representative for the organ. 

In points of the growing organ three mutually orthogonal principal growth directions (PDGs) can be recognized, unless growth is isotropic [5]. Along these directions R_l_ attains extreme values: maximal, minimal and of the saddle type. The extreme of the saddle type is the highest growth rate in a plane normal to the direction of the maximal R_l_, and at the same time the lowest growth rate in a plane normal to the direction of the minimal R_l_. These PDGs change with a position [8,13] forming PDG trajectories [14]. A pattern of PDG trajectories, considered steady, if organ geometry does not change in time, can be recognized in the cell wall system [15–17]. Two families of mutually orthogonal lines describing this system seen in a section of the organ, known as periclines and anticlines [18,19] represent PDG trajectories. This led Hejnowicz [13,14] to the hypothesis that cell divides with respect to PDGs, a division wall is typically formed in the plane perpendicular to one of PDGs at the site of its formation.

The root apex, like other plant organs, grows symplastically [1,2,19]. In angiosperms, its growth is determined by the quiescent centre (QC), i.e. the zone of a low mitotic activity [20] located at the pole of the proper root. This zone defines initial cells affecting cellular organization of the root apex [21]. In the case of Arabidopsis root, initials of particular tissues have been precisely recognized [22,23]. A diversity of cell lineages originating from them, observed in the course of intact growth and as a result of laser ablation experiments [24,25], suggests that there must be an interesting spatial and directional variation of growth rates in the very apical region of the root. However, little is known about this variation, especially in 3D. Empirical data, including those obtained by advanced computer techniques [26,27], are mostly limited to growth in one direction along the root axis, and above the quiescent centre. It remains unknown what are growth rates away from the axis, at points located in different parts of the root proper and the root cap. 

As mentioned, in order to calculate growth rates the displacement velocity field is needed. For A. thaliana root apex such field has been recently obtained [28], by combining mathematical modelling and two types of empirical data, on the published velocity profile along the root axis above QC [26] and dimensions of cell packet originated from the initials of epidermis and the root cap. One can expect that such field results from a particular distribution of the linear growth rates within the organ which has not been examined yet. What is this distribution and directional variation of R_l_ in a very apical region of the apex still becomes an open question.

The present paper aims to fill this gap. Assuming the displacement velocity field determined previously [28], it applies the GT-based modelling to generate the map of linear growth rates for the apical part of the *Arabidopsis* root. In the map the 3D indicatrices for selected points of the axial section are shown, illustrating among other things, spatial variation and directional preferences of R_l_ at positions of initial cells. Having R_l_, the degree of growth rate anisotropy is estimated in two types of planes, defined by PDGs and corresponding to exemplary cell walls recognized of the cell wall system. By **V** field modification, changes in the growth rate map are modelled.

## Materials and Methods

### Description of the root apex geometry

**Figure 2:**
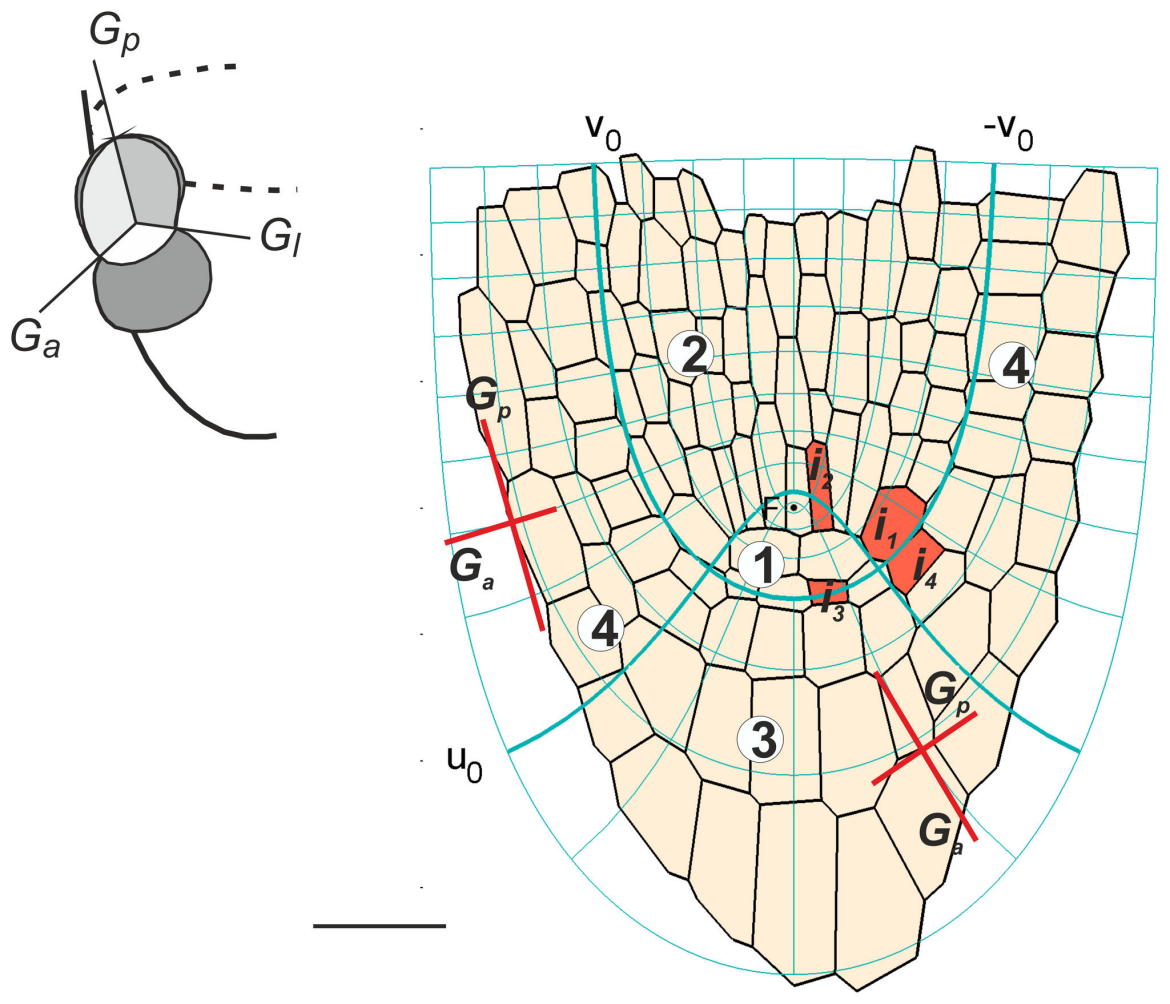
The *Arabidopsis* root apex (longitudinal section adopted from Van der Berg et al. 1998) with the root-natural coordinate system, R-NC (*u*,*v*,φ) for φ *=const*., applied to it. The exemplary initial cells (red) and two of three principal growth directions (G_a_, G_p_) are indicated; the insert shows all three PDGs in 3D. The *u* and *v* lines (thin blue) represent PDG trajectories, two of them *u*_0_ and *v*_0_ turning into -v_*0*_ (thick blue), divide the apex into four zones corresponding to: 1, 2 -the root proper without epidermis, 3, 4- the root cap with epidermis, the zone 1 represents QC. Bar = 20 µm .

Like previously [28], let us take the *Arabidopsis* root apex assumed as typical for about 1-week-old seedling [24,29]. Geometry and cell pattern of the root apex can be conveniently described in a curvilinear orthogonal coordinate system, R-NC(*u*,*v*,φ) which is natural in this sense that coordinate lines of the system represent PDG trajectories [10]. Assuming steady-state growth without a rotation around the root axis andφ*=const.* as the axial plane, the lines *u=const* and *v=const* (see also Online Resource S1 in [28]), represent anti- and ericlinal PDG trajectories, respectively (Figure 2), and the latitudinal PDG trajectories are perpendicular to this plane. The application of R-NC to the cell pattern is such that *v*_*0*_ = π/4 which turns into *-v*_*0*_ *= -π4* represents the border between the root proper and the root cap, whereas *u*_*0*_ = 0.35 represents the basal limit of the quiescent centre and the border between the columella and lateral parts of the root cap. Under this application in the root apex there are four zones representing: zone 1- the quiescent centre; zone 2 - the remaining part of the root proper without the rhizodermis; zones 3 and 4 – the columella and lateral part of the root cap with the rhizodermis, respectively. Since the root proper without the epidermis represents the Körper, while the roots cap plus epidermis represents the Kappe according to Schüpp terminology (see 19), we shall use the term Körper for zones 1and 2 and Kappe for zones 3 and 4. Notice that the root apex is symmetrical. As the coordinate system is of the confocal type, the focus situated in a topographic centre of the cell pattern happens to be within the quiescent centre (zone 1), and the root axis is represented by two lines: *v*=0 above, and *u*=0 below the focus. 

### The displacement velocities

In general, the vector **V** given in R-NC(*u*,*v*,φ) is composed of three components: V_u_, V_v_ , and V_φ_. After Hejnowicz and Karczewski [10], due to absence of rotation V_φ_= 0, we assume: V_u_= 0, V_v_= 0 in zone 1; V_u_= h_u_ c (*u*-*u*_*0*_), V_v_ = 0 in zone 2; V_u_= 0, V_v_ = -h_v_ d sin(*qv*) in zone 3; V_u_= h_u_ c *(u*-*u*_*0*_), V_v_ = - h_v_ d sin(*qv*) in zone 4, where *q*=π/*v*_*0*_, c, d are constants and h_u_, h_v_ are scale factors of the coordinate system described previously (see Text S1). It means that cells located in the zone 1 preserve their position within the root apex during growth, whereas the remaining cells grow and displace away from the quiescent centre: basipetally along *v=const* in the zone 2, acropetally along the *u=const* in the zone 3, and towards the root periphery in the zone 4. 

In accordance with the above equations, V_u_ and V_v_ depend on the parameters c and d, respectively (h_u_ and h_v_ are constant for a given position). A method of specification for these parameters was described [28]. The c=0.8 was specified on the basis of the velocity profile along the root axis above the quiescent centre [26], the d=0.12 by computer simulations in which cell packets similar to observed in the root cap were generated. 

**Figure 3:**
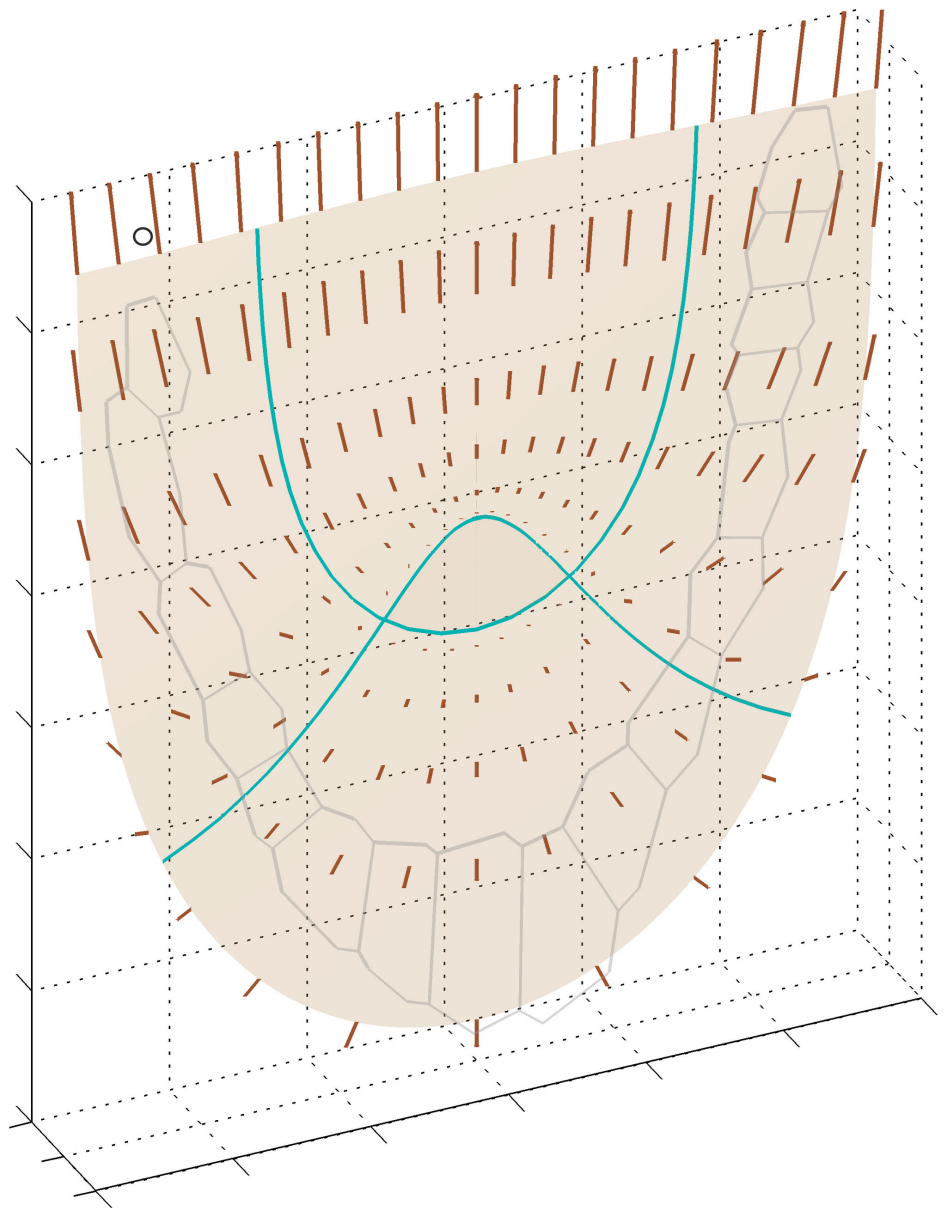
Displacement velocity field assumed for the *Arabidopsis* root apex (after Nakielski and Lipowczan 2012); in the background the outermost cell row and lines defining the root zones (see Fig. 2) are shown. The V vectors are represented by line segments, the segment indicated by circle corresponds to 0.11 µm min^-1^.

The obtained **V** field is shown in Figure 3. It can be seen that **V** vectors vary within the root apex concerning both the length and direction. In the root proper (the zone 2 plus internal part of the zone 4) the velocities increase mainly basipetally and, a little less, to the root peripheries. Within the central root cap (zone 3) the **V** vectors increase acropetally maintaining orientation tangent to the lines u=const. In the lateral root cap (external regions of the zone 4) they enlarge basipetally and into the root peripheries, whereas their orientation with respect to u=const. lines changes with position.

### Calculation of growth rates

The linear growth rate in the direction e_s_ was calculated in R-NC (*u*,*v*,φ) system using the equation [5,9]: 

$$R_{l(s)}=T_{uu}\alpha^{2}+T_{vv}\beta^{2}+T_{\phi \phi }\gamma^{2}+\left(T_{uv}+T_{vu}\right)\alpha \beta +\left(T_{u\phi }+T_{\phi u}\right)\alpha \gamma +\left(T_{v\phi }+T_{\phi v}\right)\beta \gamma$$

where T_uu_, T_vv,_ T_φφ_ and T_uv_, T_vu,_, T_uφ_,T_φu_, T_vφ_,T_φv_ are diagonal and non-diagonal respectively, components of the growth tensor matrix (see Text S1), whereas αβγ are direction cosines of e_s_. The diagonal components defined R_l_ in PDGs: T_uu_ - along e_v_, T_vv,_- along e_u_, T_φφ_ -along e_φφ_ . As the R-NC system is assumed as natural one at every point three PDGs coincide with unit vectors and, for example, in the root proper we have: G_a_= e_v_, G_p_= e_u_, G_l_ = e_φ_. The results show R_l_ indicatrices obtained for points of the axial section. A single indicatrix was drawn for R_l_ calculated for 1600 directions uniformly distributed concerning e_s_. Its orientation in 3D resulted only from directional variation of R_l_ at a given position. 

Having R_l_ for PDGs, a degree of growth rate anisotropy (DGA) for each pair of these directions, was estimated. The DGA, calculated locally as the ratio of R_l_ in two PDGs, was distributed to cells from Figure 2 and visualized in the plane defined by these directions. The cells were represented by their geometrical centres in calculations. As the ratio reaches infinity when the denominator tends to zero, the zone 1 (where no growth is assumed) was not considered. 

## Results

**Figure 4:**
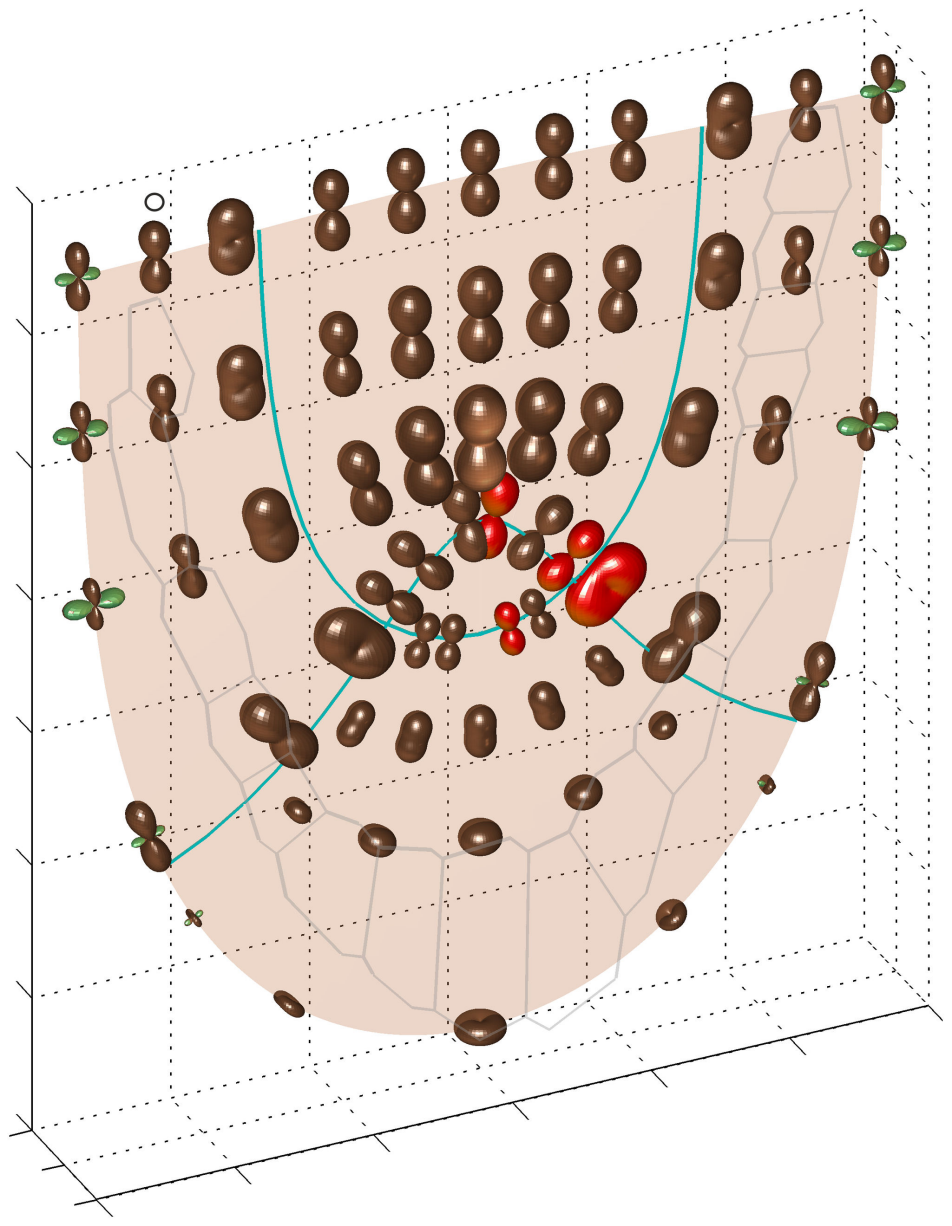
Anisotropy of growth rates in *Arabidopsis* root apex obtained for V field from Figure 3. The 3D plots show R_l_ indicatrices; those drawn in red are for initial cells (see Fig.2). In the indicatrix labelled by circle maximal R_l_ is about 8.2% h^-1^. The green plots represent negative values of the rate.

The map of linear growth rates for the *Arabidopsis* root apex is shown in Figure 4 and Movie S1. It can be seen that the rates change with both position and direction within the apex. The anisotropy of the R_l_ is evident. In the Körper (zone 2), the R_l_ in G_p_ predominates everywhere, values of the rate in two remaining PDGs are much smaller. In the Kappe (zones 3 and 4), directional preferences are not uniform. In the basal part of zone 3 there are high values of R_l_ in G_a_, but they rapidly decrease becoming minimal in the apical part of this zone. In zone 4, as in zone 2, the values of R_l_ in G_p_ are apparently the highest but there are also relatively high growth rates in G_a_, particularly, in the innermost region corresponding to the rhizodermis. These rates decrease successively with increasing distance from the root axis and finally, the contraction along G_a_ occurs (green in Figure 4) near the root cap surface where cells are shed off.

The indicatrices plotted for initial cells (red in Figure 4) are especially interesting as located in the region surrounding QC. For the initials i_1_ and i_2_ (zone 2), giving rise to the future cortex and vascular cylinder, respectively, there occur large values of R_l_ in G_p_, whereas the rates in G_a_ and G_l_ are almost completely reduced. A similar situation takes place at the position of the initial i_3,_ of the columella (zone 3), but here the direction G_a_ is dominating whereas the rates in G_p_ and G_l_ are reduced. In turn, for the initial i_4_ (zone 4), participating in the formation of the epidermis and lateral part of the root cap there is no such reduction. The highest R_l_ value is for G_p_ and the rates in G_a_ and G_l_ are three and five times smaller, respectively, in comparison to it.

**Figure 5:**
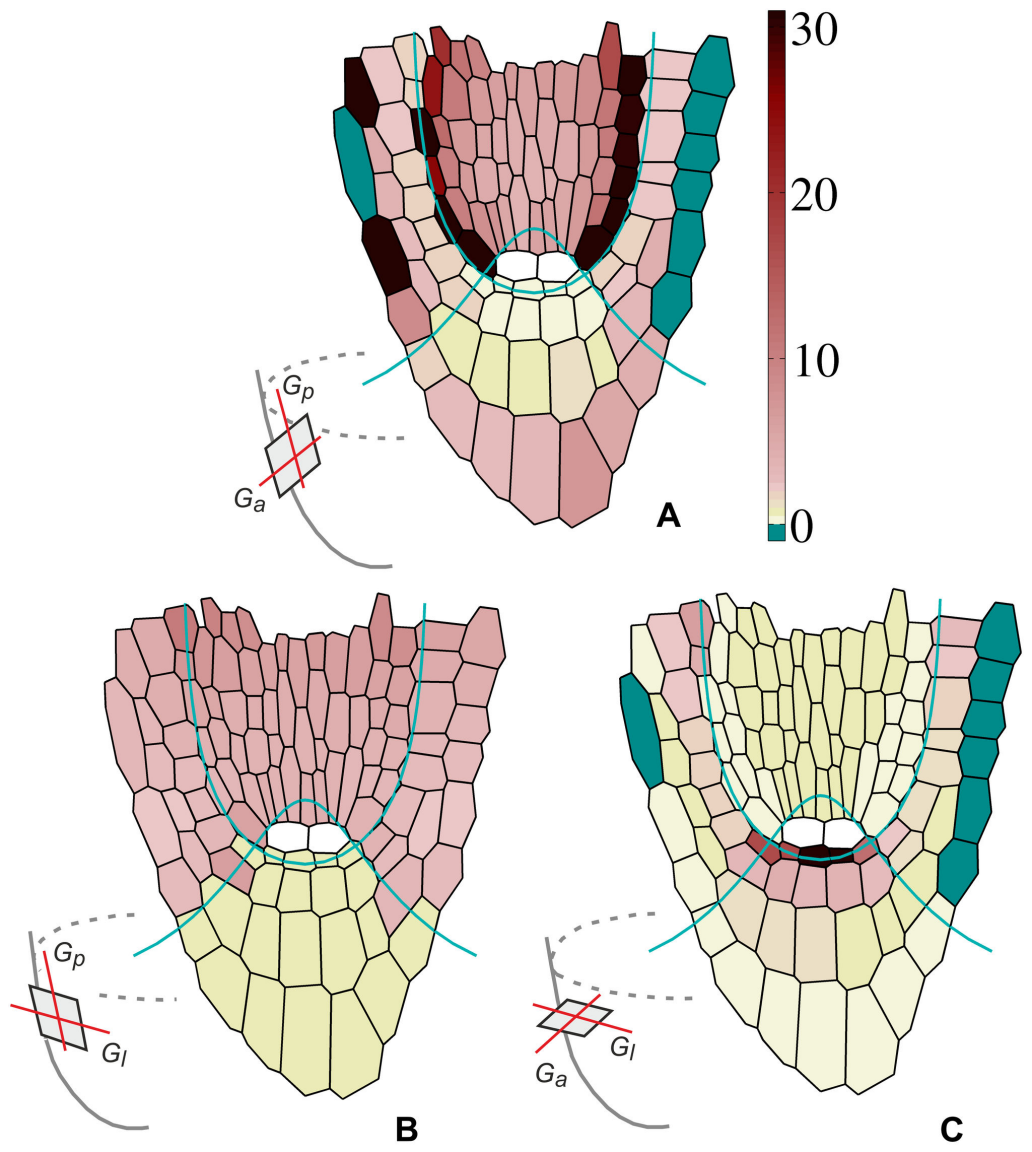
Anisotropy of growth rates in the planes defined by pairs of PDGs, visualized by the degree of growth anisotropy (DGA); the inserts show orientation of the considered planes. (A) DGA given by the ratio of R_l_ in G_p_ to R_l_ in G_a_; (B) DGA given by the ratio of R_l_ in G_p_ to R_l_ in G_l_, (C) DGA given by the ratio of R_l_ in G_a_ to R_l_ in G_l_. The DGA values are attributed to cells from Figure 2 using color-coding, the negative ones (dark green) result from compression in G_a_. For the cells localized in QC (white), the DGA has not been computed.

The variation of the degree of growth anisotropy in the planes defined by PDGs is demonstrated in Figure 5. Let us take the plane defined by G_p_ and G_a_ (Figure 5A) which is common for all cells. Excluding the quiescent centre (lack of growth) and lateral margins of the root cap (contraction along G_a_), the highest DGA values, greater than 20 are in the most external part of zone 2, whereas the lowest - in zone 3 near the quiescent centre. The planes defined by G_p_ and G_l_ (Figure 5B) which, in contrast to the previous plane change with a position, show more or less uniform distribution of DGA. The relatively high DGA values, of around 10, are in the zones 2 and 4, whereas relatively low, close to 1 (almost isotropy), in the zone 3. The planes defined by G_a_ and G_l_ (Figure 5C) also change with a position. There are rather low values of the DGA, maximal but not greater than 5 in the most internal part of the zones 3 and 4, and minimal, close to 1 - in external regions of the zones 2 and 3.

**Figure 6:**
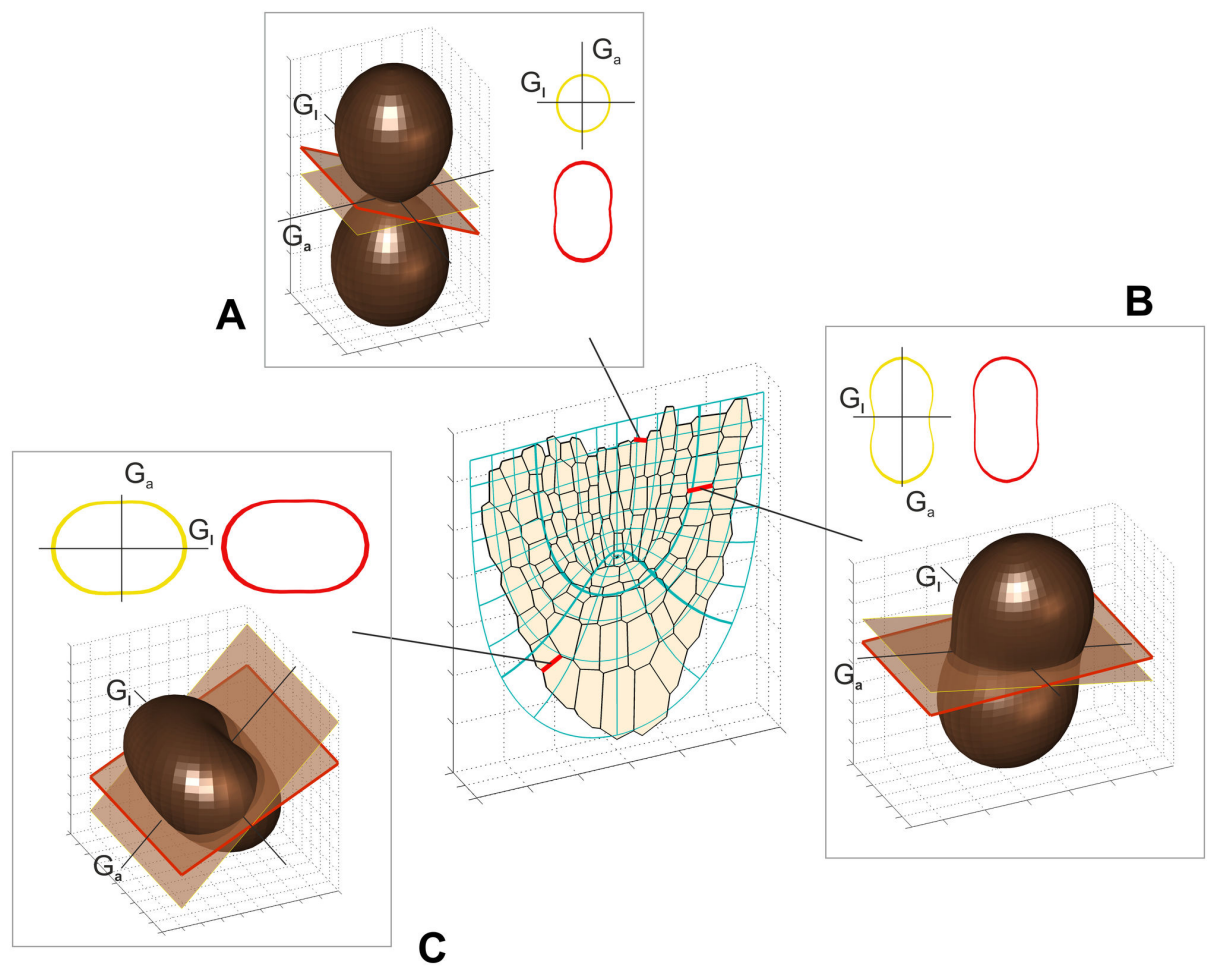
Anisotropy of growth rate in exemplary cell walls (red in the cell pattern), which are oblique with respect to PDGs. In every case, R_l_ indicatrices and their intersections by two planes: one representing the wall (red) and the other, defined by G_a_ and G_l_ (yellow), are shown. Both intersections are symmetrical with respect to these directions. The values of DGA calculated as the ratio of R_l_ in G_a_ to R_l_ in G_l_ (i.e. as in Fig. 5C) for yellow and red plots, respectively, are the following: (A) 0.92 and 2.06, (B) 1.56 and 1.62, (C) 0.44 and 0.52.

The present modelling allows one to determine R_l_ anisotropy also for the walls not lying in planes defined by PDGs. Let us consider three walls shown in Figure 6 as the example. They are oblique with respect to the PDGs, but anisotropy of R_l_ occurring in them can be conveniently interpreted in relation to the one in the plane defined by G_a_ and G_l_ (compare two intersections of the indicatrix, drawn in red and yellow). In Figure 6C where only a small difference in shape between these intersections is observed, the DGA for the wall is about 20% greater in comparison to the one for the plane defined by G_a_ and G_l._ In the case of two remaining walls where the differences in intersection shape are more pronounced, much more significant DGA increase is obtained, for the wall in Figure 6A exceeding 100 %. 

**Figure 7:**
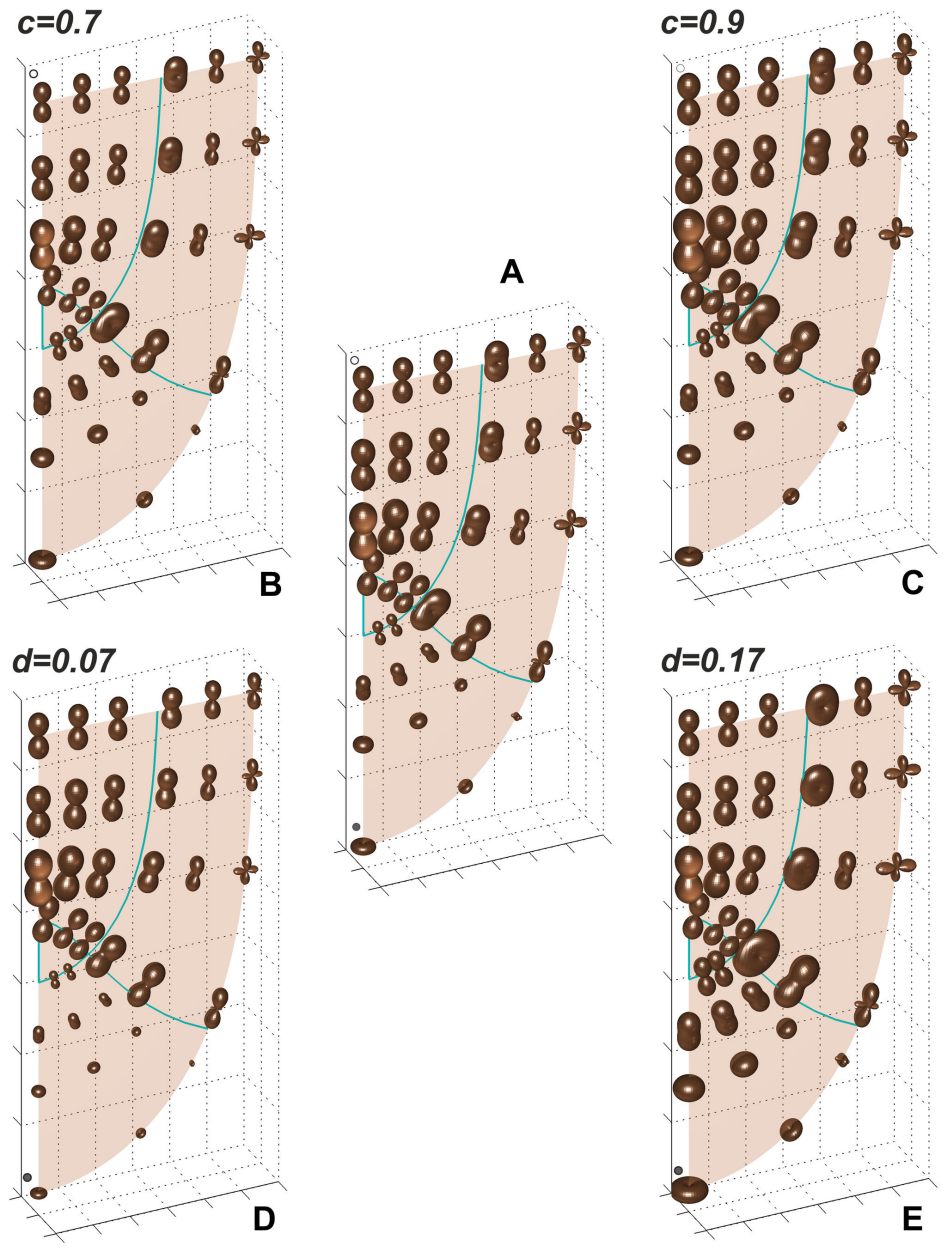
Maps of R_l_ indicatrices obtained for V field with modified values of the parameters c (B, C) and d (D, E); the map (A) corresponding to Figure 4 is a reference. The change in c leads to decrease (B) and increase (C) of R_l_ in zones 2 and 4 whereas the rates in zone 3 remain unchanged. In turn, the change in d leads to decrease (D) and increase (E) of R_l_ in zones 3 and 4 whereas the rates in zone 2 remain unchanged. The indicatrices indicated by circles are considered in the text.

**Figure 8:**
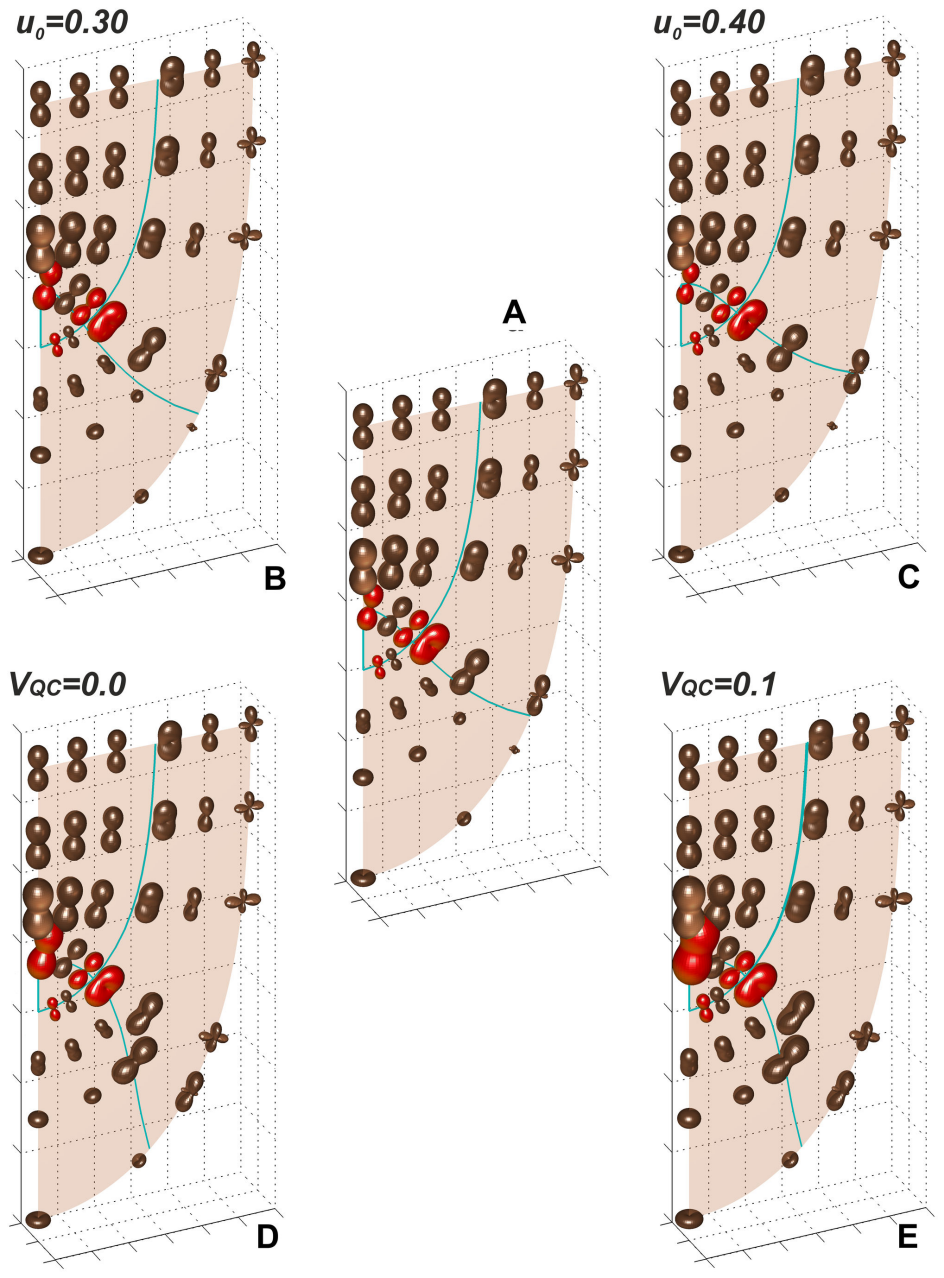
The maps of Rl indicatrices as in Figure 7 but obtained assuming modification of the proximal border of the quiescent centre (zone 1) and the central part of the root cap (zone 3); the indicatrices drawn in red are for initials from Figure 2: (A) the reference map corresponding to Figure 4 where the border is represented by the line u0=0.35; (B, C) as in (A) but for u0=0.30 and u0=0.40, respectively; (D) the border drawn on the basis of cell pattern in Figure 2; (E) as in (D) but the velocity V=0.01 m min-1 at the whole QC border is assumed.

The figures 7 and 8 visualise maps of the linear growth rates in the root apex obtained assuming changes in specification of **V**. What happens when the value of the parameter *c* is modified are shown Figure 7B,C. The changes in the zones 2 and 4 are observed in comparison to the case c=0.8 assumed here in Figure 7A and earlier in Figure 4, for c=0.7 the rates are lower, and for c=0.9 – higher than previously. A comparison of values of R_l_ in G_p_ for indicatrices indicated by open circles has shown that the rate which is equal to about 0.90 h^-1^ in Figure 7A, decreases to about 0.78 h^-1^ in Figure 7B, and increases to about 1.01 h^-1^ in Figure 7C (see also Table S1). 

The Figure 7D,E shows how the R_l_ distribution is influenced by the parameter d. As this parameter determines V_v_ the changes occur only in the root cap (zones 3 and 4), namely values of R_l_ for d=0.17 are higher, whereas for d=0.07 - smaller, comparing to those obtained for d=0.12 assumed in Figure 7A (and earlier in Figure 4). The calculations performed for indicatrices indicated by closed circle have shown that the R_l_ in G_p_ which is equal to about 0.45 h^-1^ in Figure 7A, decreases to about 0.26 h^-1^ in Figure 7D, and increases to about 0.64 h^-1^ in Figure 7E. 

The maps of R_l_ generated assuming the modification of the proximal border of the quiescent centre and the central part of the root cap are shown in Figure 8. In Figure 8A,B,C the border is represented by the line *u*_0_ of the R-NC system, for *u*_*0*_=0.30, the zones 1 and 3 are smaller, whereas for *u*_*0*_=0.40 - larger in comparison to *u*_*0*_=0.35 assumed here in Figure 8A and earlier in Figure 4. This results in modification of growth rates in both remaining zones: 2 and 4. Comparing R_l_ in G_p_ at the position of initials i_2_ and i_4,_ with reference to Figure 8A, we can see (Table S2) that in Figure 8B value of the rate increases about 9.1% for i_2_ and decreases 0.9% for i_4_. This is unlike in Figure 8C where the rate decreases about 9.0% for i_2_ and increases 0.9% for i_4_. 

The Figure 8D,E shows two cases where the considered proximal border of the quiescent centre is described not by the coordinate system, but the line drawn by hand on the basis of cell pattern in Figure 2. The difference between them depends on this that in Figure 8D, as previously there is no growth within the QC, whereas in Figure 8E such growth occurs, though small. In both, the R_l_ distribution is changed, especially in the axial region of zone 2, due to more realistic QC shape. Comparing Figure 8D to Figure 8A it can be seen (Table S2) that the maximal R_l_ at the position of the initial i_2_ increases about 27.3%, whereas values of the rate in all remaining initials remain the same. In the case in which growth in QC occurs (Figure 8E), growth rates in the whole region surrounding QC are greater than previously in Figure 8D. For example, the maximal value of R_l_ for the initials i_2_ (in G_p_) and i_4_ (in G_a_) increase 22,2% and 6.6%, respectively. In all considered cases where QC has been modified (concerning shape in Figure 8B,C,D and quiescence in Figure 8E) directional preferences of R_l_ in particular zones remain unchanged, if Figure 8A is taken as the reference.

## Discussion

### A significant anisotropy of growth rate occurs in the apical part of the root

The variation of growth rates in the apical region of *Arabidopsis* root has been modelled assuming the displacement velocity field, determined previously [28]. The results indicate that values of the linear growth rate (R_l_) change with both position within the apex and direction in which the rate is calculated. Furthermore, directional preferences of R_l_ are different in different parts of the root apex. In zone 2, which corresponds to the inner part of Körper using Schüepp terminology [19], the linear growth rate in periclinal direction (G_p_) predominates everywhere. In the Kappe (zones 3 and 4) the similar predominance occurs but only in the lateral part of the root cap (zone 4), whereas in the central region there is an increased contribution of the rate in anticlinal direction (G_a_), so that this direction becomes predominating in the basal part of the zone 3. Notice that the epidermis situated in the innermost part of zone 4 is distinguished by increased component of the R_l_ in anticlinal direction. Interestingly, this is unlike the epidermis of the shoot apex (protodermis), where because of the tunica/corpus organisation the anticlinal growth is restricted [19]. The rate along G_a_, relatively high at the innermost part of the Kappe, sharply decreases and finally attains negative values (contraction) at the lateral root cap peripheries. These negative values are very small and their occurrence, known from previous studies [10], may be associated with sloughing off of the root cap cells. Similar negative rates were found at margins of leaves, formerly in *Xanthium*, using strain rate analysis [8], lately in *Arabidopsis*, with the aid of the contemporary techniques based on digital image sequence processing [30]. 

The quiescent centre represented by zone 1 where there is no growth contrasts with surrounding regions where R_l_ values are relatively high (Figure 4). This contrast becomes deeper in both when the QC shape is more realistic (Figure 8D) and a small growth within this zone is applied (Figure 8E), in every case preserving general distribution and local preferences of growth rates in different parts of the root apex. 

The knowledge of the displacement velocities is crucial for growth variation. The present paper assumes **V** field determined previously [28], in R-NC (*u*,*v*,φ) system. As the system is curvilinear, some aspects of the relationship between **V** field (Figure 3) and R_l_ (Figure 4) need comments. Notice that V_u_ and V_v_, though defined for the axial longitudinal section, results in growth rates in all directions. Taking the zone 2 where only V_u_ occurs as the example, nonzero R_l_ values can be observed not only in e_u_ but also in many other directions in 3D, including e_v_ and e_φ_. Therefore, even under absence of V_φ_, due to lack of a rotation around the rot axis, significant nonzero growth rates in e_φ_ occur – they result from the remaining components: V_v_ in zone 2, V_u_ in zone 3, and both V_v_ and V_u_ in zone 4. The reason is that any vector considered in curvilinear coordinates depends on position via scale factors h_u_ and h_v_ of the system [28]. 

The variation of growth rates in root apices has already been studied by means of growth tensor [10] but only in general, not taking a particular species into account. Our results differ in part in comparison to those obtained previously. Here, applying *u*_*0*_=0.35 instead *u*_*0*_=0.45 and values of c and d smaller than previously, maximal linear growth rates are lower than before, both in the root proper and the root cap. In addition, remaining *v*_*0*_ the same, we include the epidermis in the zone 4, not in the zone 2, which is necessary for roots in which initials of the epidermis and the lateral root cap are common. For that reason the epidermis shows higher growth rates in anticlinal direction not observed before. These differences, important in details but not altering the global image of growth rate distribution in roots, are a result of adaptation of the general model to the case of *Arabidopsis* root apex. Other novelties of the present approach are the following: (1) the **V** field specified by empirical data is used to show anisotropy of growth rates in very apical part of the root; (2) the relationship between such field and the map of 3D indicatrices is visualized and interpreted in in terms of PDGs; (3) spatial variation of the degree of growth anisotropy is demonstrated for two types of planes, one defined by PDGs and the other, approximating real cell walls; (4) the modelling showing how the obtained growth rate variation can be modified, due to other specification of **V** field, has been developed.

### Growth rate anisotropy and orientation of cell divisions

According to Hejnowicz’s hypothesis [13,14] cells divide with respect to PDGs, a division wall lies typically in the plane defined by two PDGs, i.e., it is perpendicular to the third PDG. Both microscopic observations [15,17,31] and computer simulations [32–34] seem to support this view. In the present paper, cell divisions have not been considered. However, comparing cell pattern (Figure 2) and R_l_ map (Figure 4) we can see that cell walls of the considered *Arabidopis* root apex lie in planes defined by PDGs, at least to the first approximation. Moreover, knowing that only division walls oriented exactly perpendicular to one of PDGs (i.e. lying in the plane define by two remaining PDGs) maintain such orientation with during further growth [5,13,32], we may speculate whether they were formed according to Henowicz’s rule or not. In the root proper, for example, the walls tangent to *v=const* seem to be formed perpendicular to G_a_, and the walls tangent to *u*=const - perpendicular to G_p_. Distinguishing proliferative and formative cell divisions [23,35] one can conclude that the proliferative division result in the wall perpendicular to G_p_ in the root proper, and perpendicular to G_a_ in the root cap. The formative division, in turn, seem to be perpendicular to G_a_ or G_l_ in the root proper, whereas perpendicular and to G_p_ or G_l_ in the root cap. 

The divisions of initial cells are especially interesting. In the case of the *Arabidopsis* root apex the initials i_1_, i_2_, i_3_ (Figure 2) divide mostly transversally (proliferative division), whereas i_4_- longitudinally (formative division). These divisions interpreted with respect to PDGs are perpendicular to the direction of the maximal R_l_ , except for the initial i_4_ (Figures 4,8) where according to this rule a division tangent to *u=const* should takes place instead of tangent to *v=const*. However, Campilho et al. [29] have reported that such divisions also occur, though infrequently. 

Anisotropic expansion is an essential feature of cell walls [36,37]. If walls are typically oriented with respect to PDGs, as Hejnowicz postulated [13,14], it has been reasonable to consider the anisotropy in planes defined by pairs of these directions. Our results support the view that the degree of growth anisotropy varies with position throughout the root apex [38]. The maximal values of this degree (20-25) are in the cortex, in the planes defined by G_p_ and G_a_ (Figure 5A), minimal ones predominate for the planes defined by G_a_ and G_l_ (Figure 5C), where in some regions (yellow) growth is almost isotropic. In the root cap, in turn, the degree changes in a more complex way but rather in the range of middle and lower values. Two aspects seem to be interesting. Firstly, there are regions in which values of the degree of growth anisotropy strongly differ each other comparing the planes defined by different pairs of PDGs. For example, the external part of the zone 2 shows extremely large DGA values in the planes defined by G_p_ and G_a_, whereas small DGA values in the planes defined by G_a_ and G_l_ (Figure 5). The region of initials surrounding QC, in turn, shows relatively small DGA values in the planes defined by G_p_ and G_a_ and extremely large in the planes defined by G_a_ and G_l_. Secondly, comparing the same types of planes, the DGA calculated in the root cap changes in a relatively wide range along a short distance. Such situation occurs in the zones 3 and 4, in both going along *v=const*, from the centre of the root periphery. Whether similar properties are observed in real root apices needs detailed studies. 

In this paper the growth tensor method is applied. A determination of growth tensor field responsible for control of growth and cell divisions at the organ level is the first step of such application. This allowed us to visualize predicted spatial and directional variation of growth rate in the root apex as a whole. However, the growth process has also significant local components which must be taken into account [36]. In our approach some of them may be included as secondary factors in simulations of both growth and cell divisions. Such simulations by use of the tensor-based model for growth in which cells divide with respect to PDGs [33] are currently prepared. 

Assuming a relationship between cell size, the rate of growth, and the rate of cell divisions in 3D [39,40] the modelling can be extended on distribution of cell divisions. It would be also interesting to study growth rate anisotropy in roots that differ in velocity profiles [26] or show changes resulting from environmental conditions [40].

## Supporting Information

Text S1 **The Growth Tensor in Root-Natural Coordinate System R-NC(*u*,*v*,φ).**
(DOC)

Movie S1 **The 3D map of growth rates indicatrices for the modelled *Arabidopsis* root apex.**
(MPG)

Table S1 **Values of R_l_ along principal growth directions for selected points located at peripheries of the root apex.**
(DOC)

Table S2 **Values of R_l_ along principal growth directions for selected initial cells of the root apex.**
(DOC)
