# Supplementary material for: Spatial and Directional Variation of Growth Rates in Arabidopsis Root Apex: A Modelling Study
Source: PLoS One. 2013 Dec 18;8(12):e84337. doi: 10.1371/journal.pone.0084337 (PMC3867472; doi:10.1371/journal.pone.0084337)
Supplement: Text S1 — The Growth Tensor in Root-Natural Coordinate System R-NC(u,v,φ). (DOC) [file pone.0084337.s001.doc]

**The Growth Tensor in R-NC**(*u*,*v*,*φ*) **system**

1. Equations for R-NC(*u*,*v*,*φ*) system


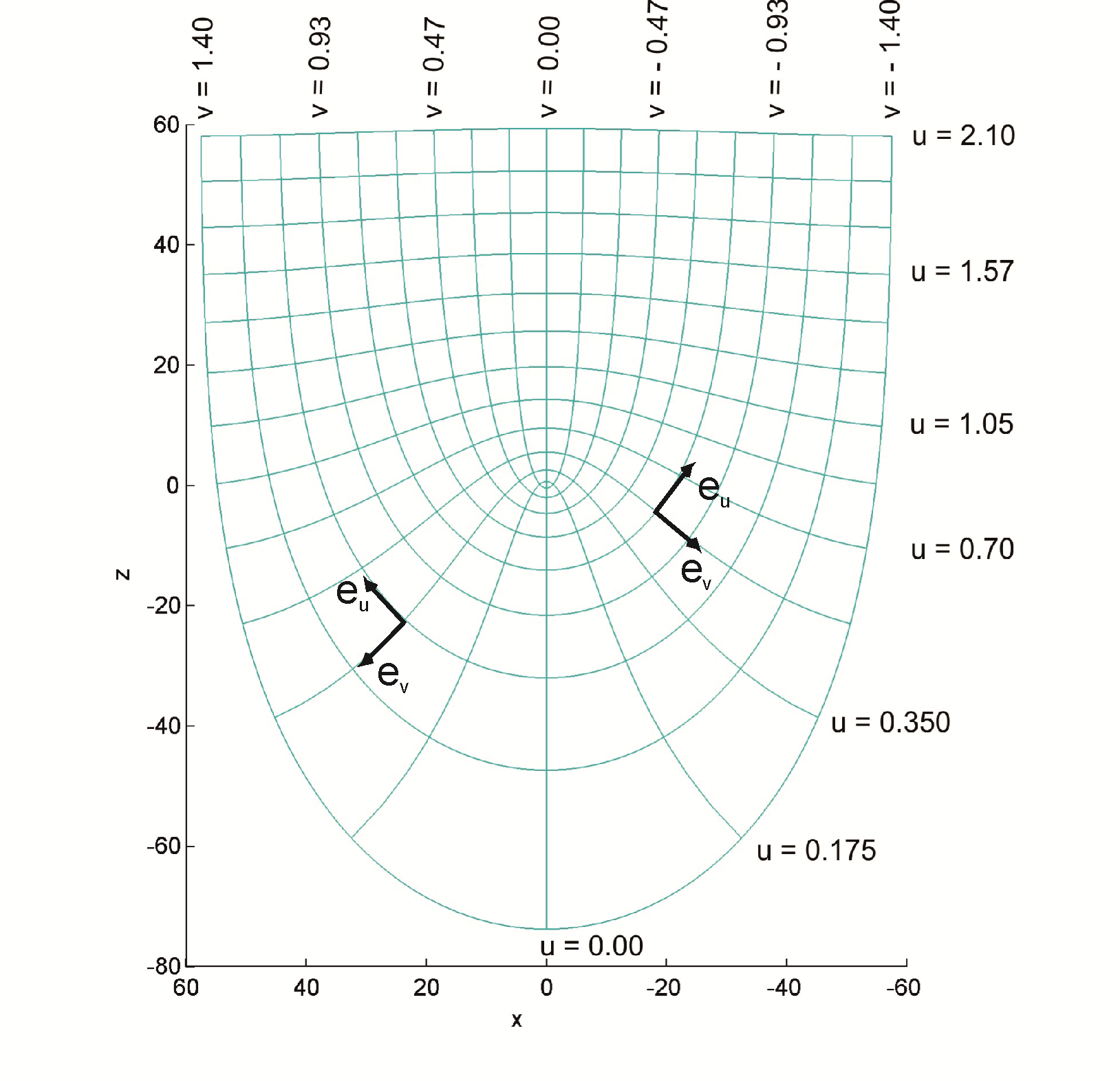


Scale factors:

$h_{u}=h_{v}=\frac{\sqrt{\sinh^{2}\left(2u\right)+\sin^{2}\left(2v\right)}}{\cosh^{2}\left(u\right)-\sin^{2}\left(v\right)}$,

$$h_{\phi}=2\arctan\left(\tanh\left(u\right)\tan\left(v\right)\right)$$

Coordinates:

$$x=\frac{2}{\pi}\arctan\left(\tanh\left(u\right)\tan\left(v\right)\right)\cos\left(\phi\right)$$

$$y=\frac{2}{\pi}\arctan\left(\tanh\left(u\right)\tan\left(v\right)\right)\sin\left(\phi\right)$$

$$z=\frac{1}{\pi}\log\left(\cosh^{2}\left(u\right)-\sin^{2}\left(v\right)\right)$$

2. The displacement velocities in R-NC(*u*,*v*,*φ*) system assumed as natural for the root apex


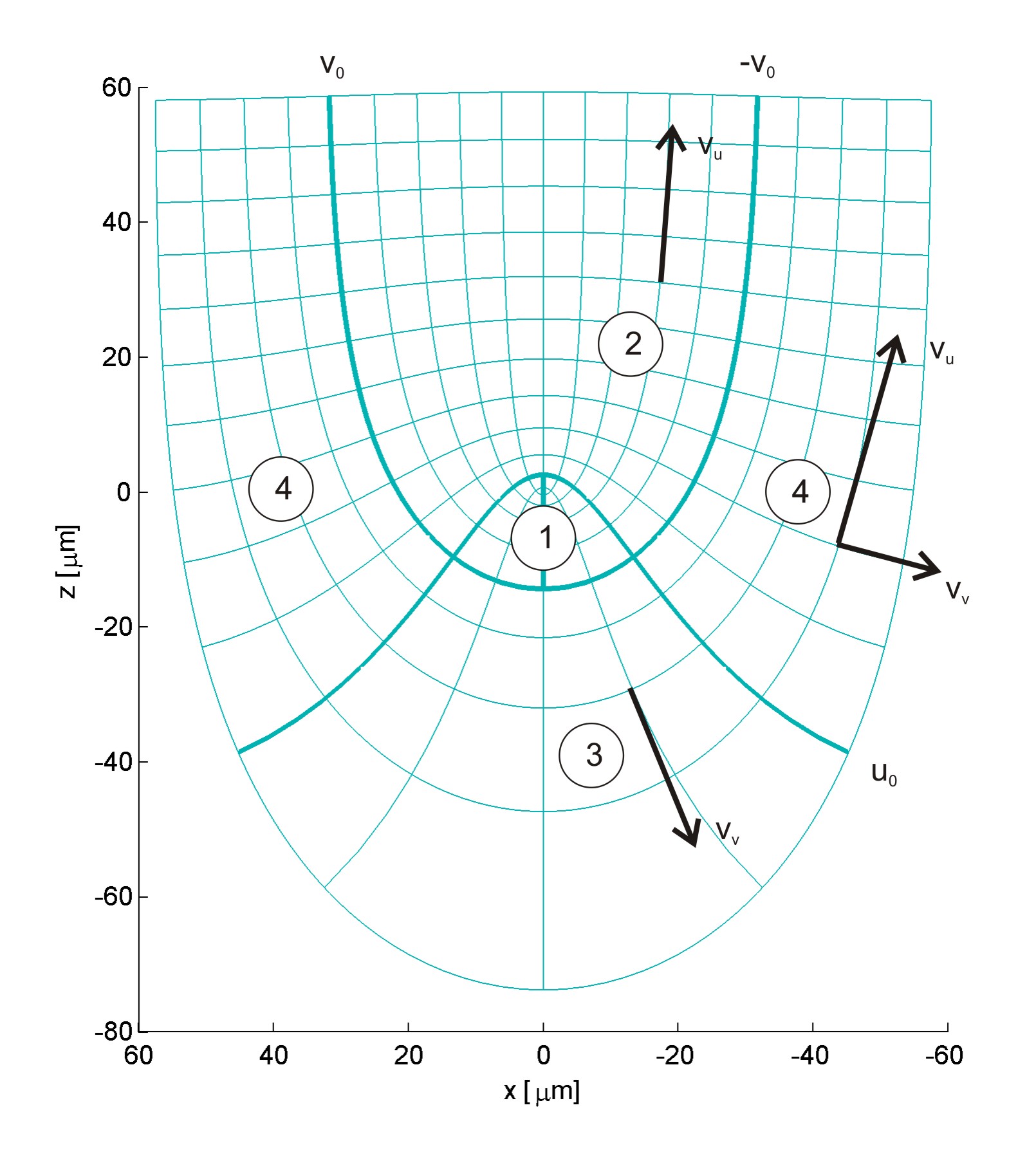


$V_{u}=h_{u}\frac{\partial u}{\partial t}$, $V_{v}=h_{v}\frac{\partial v}{\partial t}$, $V_{\phi}=h_{\phi}\frac{\partial\phi}{\partial t}$;

where

zone 1: $\frac{\partial u}{\partial t}=0$, $\frac{\partial v}{\partial t}=0$, $\frac{\partial\phi}{\partial t}=0$

zone 2: $\frac{\partial u}{\partial t}=cu$, $\frac{\partial v}{\partial t}=0$, $\frac{\partial\phi}{\partial t}=0$

zone 3: $\frac{\partial u}{\partial t}=0$, $\frac{\partial v}{\partial t}=-d\sin\left(4v\right)$, $\frac{\partial\phi}{\partial t}=0$

zone 4: $\frac{\partial u}{\partial t}=cu$, $\frac{\partial v}{\partial t}=-d\sin\left(4v\right)$, $\frac{\partial\phi}{\partial t}=0$

3. Growth Tensor:

$$GT=\left[\begin{matrix} T_{uu} & T_{uv} & T_{u\phi} \\ T_{vu} & T_{vv} & T_{v\phi} \\ T_{\phi u} & T_{\phi v} & T_{\phi\phi} \end{matrix}\right]$$

where

$$T_{uu}=\frac{1}{h_{u}}\left(\frac{\partial}{\partial u}\frac{\partial u}{\partial t}+\frac{1}{h_{v}}\frac{\partial h_{u}}{\partial v}\frac{\partial v}{\partial t}\right)$$

$$T_{uv}=\frac{1}{h_{u}}\left(\frac{\partial}{\partial u}\frac{\partial v}{\partial t}-\frac{1}{h_{v}}\frac{\partial h_{u}}{\partial v}\frac{\partial u}{\partial t}\right)$$

$$T_{u\phi}=\frac{1}{h_{u}}\frac{\partial}{\partial u}\frac{\partial\phi}{\partial t}$$

$$T_{vu}=\frac{1}{h_{v}}\left(\frac{\partial}{\partial v}\frac{\partial u}{\partial t}-\frac{1}{h_{u}}\frac{\partial h_{v}}{\partial u}\frac{\partial v}{\partial t}\right)$$

$$T_{vv}=\frac{1}{h_{v}}\left(\frac{\partial}{\partial v}\frac{\partial v}{\partial t}+\frac{1}{h_{u}}\frac{\partial h_{v}}{\partial u}\frac{\partial u}{\partial t}\right)$$

$$T_{v\phi}=\frac{1}{h_{v}}\frac{\partial}{\partial v}\frac{\partial\phi}{\partial t}$$

$$T_{\phi u}=\frac{1}{h_{\phi}}\left(\frac{\partial}{\partial\phi}\frac{\partial u}{\partial t}-\frac{1}{h_{u}}\frac{\partial h_{\phi}}{\partial u}\frac{\partial v}{\partial\phi}\right)$$

$$T_{\phi v}=\frac{1}{h_{\phi}}\left(\frac{\partial}{\partial\phi}\frac{\partial v}{\partial t}-\frac{1}{h_{v}}\frac{\partial h_{\phi}}{\partial v}\frac{\partial\phi}{\partial t}\right)$$

$$T_{\phi\phi}=\frac{1}{h_{\phi}}\left(\frac{\partial}{\partial\phi}\frac{\partial\phi}{\partial t}+\frac{1}{h_{u}}\frac{\partial h_{\phi}}{\partial u}\frac{\partial u}{\partial t}+\frac{1}{h_{v}}\frac{\partial h_{\phi}}{\partial v}\frac{\partial v}{\partial t}\right)$$

Principal growth directions are represented by unit vectors of the system. The relative elemental rates of growth along these directions are given by diagonal elements of GT matrix:

$$R_{l\left(e_{u}\right)}=T_{uu}$$

$$R_{l\left(e_{v}\right)}=T_{vv}$$

$$R_{l\left(e_{\phi}\right)}=T_{\phi\phi}$$
