# Supplementary material for: Spatial and Directional Variation of Growth Rates in Arabidopsis Root Apex: A Modelling Study
Source: PLoS One. 2013 Dec 18;8(12):e84337. doi: 10.1371/journal.pone.0084337 (PMC3867472; doi:10.1371/journal.pone.0084337)
Supplement: Table S1 — Values of Rl along principal growth directions for selected points located at peripheries of the root apex. (DOC) [file pone.0084337.s003.doc]

**Table S1**. Values of R_l_ [% h^-1^] in each of three principal growth directions obtained for selected points in Fig.7 (indicatrices indicated by circles). The A^0^, B^0^, C^0^ correspond to figures 7A, 7B,7C (open circles) , whereas the A_0_, D_0_, E_0_ to figures 7A, 7D, 7E (close circles), respectively.

|  | R_l(Gp)_ | R_l(Ga)_ | R_l(Gl)_ |
| --- | --- | --- | --- |
| A^0^ | 9.384 | 1.012 | 1.012 |
| B^0^ | 8.210 | 0.885 | 0.885 |
| C^0^ | 10.557 | 1.139 | 10.557 |
| A_0_ | 4.733 | 0.837 | 4.733 |
| D_0_ | 2.761 | 0.489 | 2.761 |
| E_0_ | 6.705 | 1.186 | 6.705 |
