# Supplementary material for: Spatial and Directional Variation of Growth Rates in Arabidopsis Root Apex: A Modelling Study
Source: PLoS One. 2013 Dec 18;8(12):e84337. doi: 10.1371/journal.pone.0084337 (PMC3867472; doi:10.1371/journal.pone.0084337)
Supplement: Table S2 — Values of Rl along principal growth directions for selected initial cells of the root apex. (DOC) [file pone.0084337.s004.doc]

**Table S2**. Values of R_l_ [% h^-1^] in each of three principal growth directions obtained for initial cells in Fig.8 (indicatrices plotted in red). The A, B, C, D, E, correspond to figures 8A, 8B, 8C, 8D, 8E, respectively.

|  | | R_l(Gp)_ | R_l(Ga)_ | R_l(Gl)_ |
| --- | --- | --- | --- | --- |
| A | i_1_ | 8.451 | 0.080 | 0.956 |
|  | i_2_ | 9.299 | 0.928 | 1.020 |
|  | i_3_ | 0.143 | 5.158 | 0.145 |
|  | i_4_ | 8.974 | 5.270 | 1.728 |
| B | i_1_ | 8.522 | 0.151 | 1.825 |
|  | i_2_ | 10.142 | 1.771 | 1.947 |
|  | i_3_ | 0.143 | 5.158 | 0.145 |
|  | i_4_ | 8.894 | 5.190 | 2.543 |
| C | i_1_ | 8.379 | 0.007 | 0.087 |
|  | i_2_ | 8.456 | 0.085 | 0.093 |
|  | i_3_ | 0.143 | 5.158 | 0.145 |
|  | i_4_ | 9.054 | 5.349 | 0.911 |
| D | i_1_ | 8.451 | 0.080 | 0.956 |
|  | i_2_ | 11.829 | 3.457 | 3.802 |
|  | i_3_ | 0.143 | 5.158 | 0.145 |
|  | i_4_ | 8.974 | 5.270 | 1.728 |
| E | i_1_ | 8.553 | 0.181 | 2.189 |
|  | i_2_ | 14.450 | 6.079 | 6.684 |
|  | i_3_ | 1.141 | 6.155 | 1.156 |
|  | i_4_ | 9.564 | 5.860 | 3.426 |
